# Enhanced science–stakeholder communication to improve ecosystem model performances for climate change impact assessments

**DOI:** 10.1007/s13280-014-0553-4

**Published:** 2014-09-20

**Authors:** Anna Maria Jönsson, Olle Anderbrant, Jennie Holmér, Jacob Johansson, Guy Schurgers, Glenn P. Svensson, Henrik G. Smith

**Affiliations:** 1 Department of Physical Geography and Ecosystem Science, Lund University, Sölvegatan 12, 223 62 Lund, Sweden; 2 Department of Biology, Lund University, Sölvegatan 37, 223 62 Lund, Sweden; 3 Centre for Environmental and Climate Research, Lund University, Sölvegatan 37, 223 62 Lund, Sweden; 4 Department of Geosciences and Natural Resource Management, University of Copenhagen, Øster Voldgade 10, 1350 Copenhagen, Denmark

**Keywords:** Sustainable management, Agriculture, Forestry, Nature conservation, Adaptation strategies

## Abstract

In recent years, climate impact assessments of relevance to the agricultural and forestry sectors have received considerable attention. Current ecosystem models commonly capture the effect of a warmer climate on biomass production, but they rarely sufficiently capture potential losses caused by pests, pathogens and extreme weather events. In addition, alternative management regimes may not be integrated in the models. A way to improve the quality of climate impact assessments is to increase the science–stakeholder collaboration, and in a two-way dialog link empirical experience and impact modelling with policy and strategies for sustainable management. In this paper we give a brief overview of different ecosystem modelling methods, discuss how to include ecological and management aspects, and highlight the importance of science–stakeholder communication. By this, we hope to stimulate a discussion among the science–stakeholder communities on how to quantify the potential for climate change adaptation by improving the realism in the models.

## Introduction

On-going climate change will have profound consequences for forestry and agriculture, affecting both production and environmental quality. A warmer climate will prolong the growing season at northern latitudes, which can have positive effects on biomass production. However, this may be offset by more frequent attacks by pests and pathogens and an increased risk of extreme weather events (Olesen et al. 2011). Climate change will also alter the natural distribution of species, with potentially negative effects on biodiversity (Parmesan 2006) and ecosystem functioning (Walther 2010). It may also indirectly cause land-use changes driven by expansion and intensification of forestry and agriculture as a result of increased demands for food, fibre and biofuels, potentially exacerbating both the spread of insect pests and loss of biodiversity by fragmentation and loss of natural habitats (Lawler 2009). Additionally, land-use changes, whether caused indirectly by climate change or resulting from other drivers, may worsen the climate-related risks by e.g. affecting species’ abilities to shift ranges or evolve in response to climate change.

To develop adaptation and mitigation strategies, it is essential to understand the combined effects of changes in climate and land use on ecosystem structure and functioning (González-Varo et al. 2013). Linking empirical studies with ecosystem modelling and global change scenarios makes it possible to disentangle complex cause and effect relations and make predictions beyond the range of historical experience, to provide new insights on ecosystem resilience and the effect of different management strategies. All models are however simplifications and the process of model development includes prioritizing among aspects to incorporate. In this paper we give a brief overview of different modelling methods, discuss the need of including ecological interactions and management aspects, and highlight the importance of science–stakeholder communication.

## The need for process-based impact models

A wide range of model types have been developed to represent our understanding of ecosystem response to changes in environmental conditions caused by direct or indirect effects of climate change. We here use the general term “impact models” for process-based models designed to simulate climate change effects, using land-use scenarios and climate model data as input. The biotic parameters are responding to weather variables by mechanistic descriptions of the major processes involved. These models thereby differ from statistical models that describe observed covariation by empirical functions. Choosing a process-based model, rather than an empirical model, is particularly important when assessing biotic response to conditions beyond observations, e.g. climate change, and for studying ecosystem response to simultaneous changes in environmental factors such as temperature, precipitation, radiation, CO_2_ concentration, and air pollution.

Some impact models focus on species-specific responses and others on ecosystem structure and functioning. Dynamic global vegetation models (DGVMs) capture the large-scale impact of climate on terrestrial ecosystems (Ostle et al. 2009). Crop growth models focus on the annual growth and development of main agricultural crops, and have been developed and applied to predict crop yields and assess effects of changes in agricultural policy and practice (Bouman et al. 1996). In recent years parts of the functionality of crop growth models have been adopted for use in vegetation models to assess future crop distributions (Ciais et al. 2011; Van den Hoof et al. 2011). DGVMs have in turn been merged with climate models (Ostle et al. 2009), forming so called Earth system models, to assess the feedbacks between terrestrial ecosystem functions and climate change.

Both DGVMs and crop models are predominantly process-based, since mechanistic model descriptions are more likely to capture the complex interplay of various factors and better represent our understanding of plant functioning than statistical models. The distinction between mechanistic models and empirical methods is however not a black-and-white contrast. Whereas some processes are understood in great detail and can be described mechanistically, others are less well-understood or simply too complex to summarize in simple expressions that are valid under all simulated conditions. The photosynthesis is an example of a well-described process. It is however still influenced by knowledge gaps, e.g. the acclimation of photosynthesis to changes in climate conditions is poorly understood (Gunderson et al. 2010). In practice, a purely mechanistic model does not exist, because it will always rely on model parameters obtained from laboratory or field studies. Also, it may not be feasible to include detailed information about species-specific responses to microclimatic conditions, e.g. insect development as a function of host plant temperature, if it does not correspond to the accuracy of the climate model data used as input to the model simulations.

## Model simulations to support decision making

To quantify the long-term impact of climate change, impact model simulations representing a range of climate scenarios and management alternatives have to be considered. That is, a comparison between multiple model runs is needed to identify cumulative effects, thresholds and tipping points, as well as the potential to influence the outcome. Since the production and interpretation of multiple model runs is time consuming, a science–stakeholder dialogue that pin-points the research questions in terms of implementable alternative management regimes, can be very useful. In agricultural applications, it may be of relevance to compare different crops, sowing dates, application of pesticides, and timings of harvest. In a forestry context, the model simulations may include tree species selection at regeneration, different thinning regimes and timings of final harvest, as well as salvage and sanitary cutting in response to disturbances (Jönsson et al. 2013). Moreover, scenario descriptions of landscape properties and land use are useful when modelling ecosystem dynamics and interactions among species.

When developing the simulation strategy, it is important to recognise differences between forest and agricultural ecosystems, as well as between intensively managed ecosystems and nature protection areas, in terms of resistance to climate change and resilience to disturbances. Production forest stands have decadal to centennial rotation periods, unless managed as a continuous cover forest, and decisions for shaping the forest are made at regeneration and thinning, whereas agricultural management can change on a short-term basis through selection of new crop rotations or amounts of agricultural inputs in the form of e.g. inorganic fertilizers and pesticides. However, maintaining functional biodiversity in managed as well as protected areas requires a long-term (centennial) commitment, and model simulations considering both climate change and changes in land use could offer valuable insights into ecosystem dynamics and species-specific vulnerability (Gillson et al. 2013).

Choosing tree species, agricultural crops and management strategies with lower risks is often regarded as a way forward in climate change adaptation. Intensive and expensive damage prevention can, however, have negative ecosystem effects and put constrains on the private as well as public economy. The decision process then becomes less straight forward, and dependent on climate variability, i.e. the calculated risk. That is, the incentive to grow a crop susceptible to high or low temperatures may depend on the expected economic return in a climate transition period, and the benefit of carrying out intensive countermeasures against an insect pest or pathogen could depend on the climate-dependent probability of establishment of a permanent population. If the increase in risk is modest, or the potential to influence the risk is considered to be low, it may be a better option to develop a strategy on how to react if damage occurs.

A benefit of impact models is that they can be integrated into decision frameworks (e.g. agent-based models) where uncertainty stemming from different sources (Polasky et al. 2011; Robertson et al. 2013) can be handled and the preferences of different management alternatives can be studied. In addition, process-based models can be programmed to simulate the effect of lack of information, useful for identifying robust countermeasures and adaptation strategies that will fulfil the goals regardless of uncertainties (Carrasco et al. 2010).

## Science–stakeholder dialogues

Stakeholders such as landowners, practitioners, and officials at regional and national administrations are often the receivers of scientific findings. However, stakeholders should not only be viewed as receivers of the final product; instead science–stakeholder interactions are fruitful in all stages of the research process, from problem formulation to the evaluation of results. This is true also for the development of impact models that will benefit from in-depth understanding of critical questions concerning the land use (Littell et al. 2011). That is, impact models are commonly used for assessing changes in potential production, but a comprehensive evaluation of climate change effects can include a wide range of aspects such as management strategies, risk taking, expected economic outcome, biodiversity effects, energy consumption and emission of greenhouse gases. In addition to provide a reality check for the research, science–stakeholder dialogues can make knowledge and data available that otherwise would remain unknown or difficult to access (Welp et al. 2006).

To highlight the multitude of economic, social and ecological goals in managed landscapes, it is of importance that stakeholders representing all relevant perspectives are included in the research process. In this respect, one purpose of the research is to raise awareness about potential goal conflicts among stakeholders, e.g. private and public organisations (Welp et al. 2006). An identification of the stakeholder community should therefore be carried out in the initial stage of a research process (Reed 2008). General information about stakeholder preferences could be gathered by interviews or surveys. To get an expert opinion on policy options and management alternatives, however, a closer interaction with stakeholders highly relevant to the research question is needed (Phillipson et al. 2012). Officials and advisors at governmental organizations and companies are commonly involved as experts. Studies related to forestry and agriculture can benefit from also including land owners, since they are the final decision makers that will be directly affected by the consequences. Private land owners, as well as non-governmental organizations and private persons (i.e. consumers), can provide useful input to the modelling process, for instance by contributing to scenario-narratives (Volkery et al. 2008; Gillson et al. 2013). The science–stakeholder interaction may also include feedback on research results in terms of an extended peer review as a test for social robustness (Hage et al. 2010; Petersen et al. 2011). Once the model result is available, it can be used as support for stakeholders to make decisions, sort out conflicts and agree on responsibilities in a process separated from the research process. The production of knowledge is, however, an iterative process, which will benefit from a continued science–stakeholder dialogue (Welp et al. 2006; Petersen et al. 2011).

## Making impact models more ecologically realistic

Vegetation models and crop models are commonly designed to simulate the potential production, and a science–stakeholder dialogue focusing on key ecological processes is very important, since most impact models need development to provide more realistic estimates on production. The effects of abiotic and biotic stress factors, as well as land use and alternative management strategies, have to be included as process-based descriptions of e.g. fire, storm, herbivores, weeds, pests and pathogens (Soussana et al. 2010; Seidl et al. 2011). To initiate a science–stakeholder dialogue aiming at identifying impact model weaknesses and potential to provide specific decision support we suggest that the following questions should be addressed: Which are the ecosystem services of particular interest to the organisation that you represent? Have you experienced any apparent conflicts between biomass production and environmental considerations? Which spatial and temporal resolution is needed for the model to provide useful decision support? Which ecosystem processes, species interactions and adaptation strategies should be included for the model to provide reliable results?

Regulatory mechanisms like habitat size, trophic structure, intra- and inter- specific competition for resources, density-dependent responses, and evolutionary feedback mechanisms are well acknowledged in the field of ecology, but they are rarely included in projections of future climate impacts. These mechanisms pose particular challenges to future projections as they can cause non-linearities in perturbation-response relationships. A central question to climate change impact assessments, tightly linked to the issue about ecological realism, is the effect caused by inter-annual variation in weather conditions. The extreme events associated with high risk can have a large influence on both tree growth and crop production, and can thus have a critical influence on the decision making process (Reyer et al. 2013). Inter-annual variations also influence the interaction between species, and climate impact assessments should consider both climatological limitations and landscape properties influencing dispersal and migration of insect pests and fungal pathogens.

For insect pests, modelling of climate-dependent phenology and potential distribution is more common than modelling of inter-annual population dynamics. When modelling insect phenology, knowledge of the influence of weather conditions on timing of reproduction, development of the new generation and winter survival is required. Modelling of the population dynamics requires additional information on host plant response to changes in climate conditions, since the host influences the survival and reproduction of associated pests. Time-series of monitoring data for pests and pathogens can provide valuable information for model development, but since the trend over time commonly indicates climate effects as well as changes in management practise it can be difficult to separate causes and effects (Scherm 2004). The species-specific ecology and evolutionary history can help to identify processes influencing the distribution range and the species-specific potential to invade new areas or adapt to environmental changes (Lyytinen et al. 2008). In addition, evolutionary principles can form the conceptual basis for a large range of predictive models with relevance for agriculture, including changes in host-pathogen dynamics (Thrall et al. 2011).

Current models rarely include any trophic interactions (Urban et al. 2013). Natural enemies are, however, important in pest control, and should therefore always be considered as a potential model component in impact studies (Harmon et al. 2009). Climate change can influence the geographical distribution, timing of activity and developmental rate, and thereby cause spatial and/or temporal shifts in the occurrence of insect pests and natural enemies, which influence the effect of biological control (Thomson et al. 2010). Even if a species is positively affected by climate change, its enemies may also be favoured (Freier et al. 1996). Another aspect is that the viability of newly established populations will be influenced by the potential escape from natural enemies (Pelissie et al. 2010; Roos et al. 2011).

Model simulations of future distribution ranges of different plant and insect species are associated with uncertainties, which may make it difficult to decide on adaptive measures. One reason is that knowledge on what is restricting the realized distribution in comparison with the potential climate limited distribution is commonly lacking (Ulrichs and Hopper 2008). Furthermore, parasites of insect pests are influenced by the spatial distribution of food resources (plants) and host insects (for reproduction) in relation to each other (Banks et al. 2008). The challenge is to understand how the occurrence of different species is influenced by climatic factors, biotic interactions, species-specific dispersal and migration behaviour (Heikkinen et al. 2006), and to incorporate this knowledge in a process-based model for making future projections. Uncertainties can then be handled by identifying management options that result in tolerable outcomes (Burgman et al. 2005).

## Communicating model uncertainties

Incomplete and imperfect process descriptions are important sources of uncertainty, influencing both impact models and the underlying projections obtained from climate models. An impact assessment that does not handle uncertainties can be misleading. However, the science–stakeholder communication is often impaired by the fact that all model simulations come along with uncertainties. One of the main purposes with the science–stakeholder dialogue is therefore to reduce uncertainties, in particular those associated with the impact model structure. Furthermore, to improve clarity as to what the decision support represents in relation to a wide range of potential future developments, it is important to specify why, how and to what extent the results are uncertain, i.e. the source, nature and level of uncertainty (Refsgaard et al. 2013).

Knowledge gaps create uncertainties in model parameterisations, and failure to represent important processes can cause model biases. These kinds of uncertainties (epistemic) are usually handled by comparing data from several climate models, i.e. ensemble simulations (Semenov and Stratonovitch 2010). It can also be useful to carry out ensemble simulations with impact models (Challinor et al. 2009), since all models have their own history of development in terms of original research question, departmental expertise, past knowledge level and modelling tool (Colbach 2010). Bias correcting methods are commonly applied to global and regional climate model data, but it is generally difficult to interpret the effect in terms of uncertainty reduction (Ehret et al. 2012). Different species have different environmental requirements, which in turn influence how sensitive a specific impact study will be to uncertainties in climate data (Chokmani et al. 2001). Knowledge uncertainties can however be addressed by designed model experiments and sensitivity analysis, useful for identifying areas where targeted experimental research can improve model performance (Yonow et al. 2004).

A driving force in the development of impact models is the concurrent development of climate models leading to higher temporal and spatial resolutions and improved representation of weather and climate extremes (Christensen et al. 2009). Greenhouse gas emission scenarios (Nakicenovic and Swart 2000) and representative concentration pathways (Moss et al. 2010) have been established for model projections of climate change, representing the genuine (non-reducible) uncertainties about the future global development and its impact on the greenhouse gas concentrations. Effects of climate change on global land use and trade are inherently difficult to predict, and climate change scenarios commonly have to be combined with land-use scenarios to address questions about ecosystem response (de Chazal and Rounsevell 2009). Also the process of decision-making and selection of adaptation strategies generates uncertainties, stemming from goal conflicts among stakeholders that cannot easily be solved (Reilly and Willenbockel 2010). To take this value uncertainty into account, it is essential that the science–stakeholder dialogues include a multitude of perspective, and involve also actors outside the traditional agricultural and forestry sectors.

## Conclusions

The production of knowledge benefits from a science–stakeholder dialogue that makes use of the tension between societal need of concrete decision support and scientific exploration of unknowns in an iterative way. Impact models have been developed to make future projections on ecosystem functioning and productivity, both on the global and regional scale, and the model projections are used by decision makers to develop adaptation strategies. Current impact model projections do however often not address important ecological feed-back mechanisms. Species-specific impact models are commonly used without taking the population dynamics, including trophic interactions, into account, and separate modelling of vegetation growth and insect pest development makes it difficult to assess the risk of damage. Few studies address management options and decision making, including economic considerations. In addition, current impact assessments do commonly not handle uncertainties associated with the used climate model data. Great simplifications are commonly made, such as presenting results of model simulations in relation to average climate conditions rather than inter-annual variations in weather conditions. It is often not clear to the stakeholders how the research findings link to management options, and what the decision support represents in relation to different future scenarios. We therefore argue for an improved science–stakeholder collaboration to link empirical studies and impact modelling with policy and strategies for sustainable management. The central goals of such activities are to identify model weakness in terms of un-incorporated variables that represent key drivers of the ecosystem processes, discuss uncertainties of model projections in relation to management options, and find ways to fill important knowledge gaps.
